# Frequency-specific genetic influence on inferior parietal lobule activation commonly observed during action observation and execution

**DOI:** 10.1038/s41598-017-17662-x

**Published:** 2017-12-15

**Authors:** Toshihiko Araki, Mai Onishi, Takufumi Yanagisawa, Masayuki Hirata, Yoshiyuki Watanabe, Soshiro Ogata, Kazuo Hayakawa, Chika Honda, Mikio Watanabe, Yoshinori Iwatani, Shiro Yorifuji

**Affiliations:** 1 Division of Functional Diagnostic Science, Graduate School of Medicine, Osaka University, Osaka, 565-0871 Japan; 2 Department of Medical Technology, Osaka University Hospital, Suita, Osaka, 565-0871 Japan; 3 Division of Clinical Neuroengineering, Global Center for Medical Engineering and Informatics, Osaka University, Osaka, 565-0871 Japan; 4 Department of Neurosurgery, Graduate School of Medicine, Osaka University, Osaka, 565-0871 Japan; 5 Department of Diagnostic and Interventional Radiology, Graduate School of Medicine, Osaka University, Osaka, 565-0871 Japan; 6 Department of Health Promotion Science, Graduate School of Medicine, Osaka University, Osaka, 565-0871 Japan; 7 Japan Society for the Promotion of Science, Tokyo, Japan; 8 Mie Prefectural College of Nursing, Tsu, Mie 514-0116 Japan; 9 Center for Twin Research, Graduate School of Medicine, Osaka University, Osaka, 565-0871 Japan; 10 Division of Biomedical Informatics, Graduate School of Medicine, Osaka University, Osaka, 565-0871 Japan

## Abstract

Brain activity relating to recognition of action varies among subjects. These differences have been hypothesised to originate from genetic and environmental factors although the extent of their effect remains unclear. Effects of these factors on brain activity during action recognition were evaluated by comparing magnetoencephalography (MEG) signals in twins. MEG signals of 20 pairs of elderly monozygotic twins and 11 pairs of elderly dizygotic twins were recorded while they observed finger movements and copied them. Beamformer and group statistical analyses were performed to evaluate spatiotemporal differences in cortical activities. Significant event-related desynchronisation (ERD) of the β band (13–25 Hz) at the left inferior parietal lobule (IPL) was observed for both action observation and execution. Moreover, β-band ERD at the left IPL during action observation was significantly better correlated among monozygotic twins compared to unrelated pairs (Z-test, *p* = 0.027). β-band ERD heritability at the left IPL was 67% in an ACE model. These results demonstrate that β-band ERD at the IPL, which is commonly observed during action recognition and execution, is affected by genetic rather than environmental factors. The effect of genetic factors on the cortical activity of action recognition may depend on anatomical location and frequency characteristics.

## Introduction

Recognizing action is crucial for humans^1–3^. Humans imitate the actions of others and can understand the intentions of others by cognitively processing observations of action^4–6^. Despite its importance, there are individual differences in the ability to observe action^7^. It is hypothesised that these differences are caused by diversity in the brain’s activity due to genetic and environmental factors^8,9^. In fact, brain activity during action observation is influenced by the acquired motor skills of the observer^10^. However, how genetic and environmental factors influence brain activity during action observation remains unclear.

Twin studies have been conducted to estimate genetic and environmental influences. Comparisons of monozygotic and dizygotic twins are useful for investigating genetic and environmental effects, assuming that monozygotic twins share 100% of their genes, whereas dizygotic twins on average share only half of their segregating genes. In particular, elderly twins are good subjects to investigate the genetic and environmental effects on brain activity, because long-term differences in living environments experienced after twins aged and started living separately may have caused differences in brain function. If monozygotic twins are more similar in a trait than dizygotic twins, we can speculate that the trait is influenced by genetic rather than environmental factors.

Previous twin studies have reported similarities in brain activity between monozygotic and dizygotic twins using electroencephalography (EEG)^11,12^, magnetoencephalography (MEG)^13,14^ and functional magnetic resonance imaging (fMRI)^15,16^. A comparison of EEGs in twins demonstrated that much of the variance in EEG power during the resting state is explained by genetic factors^17^. Similarly, MEG studies involving twins demonstrated that genetic factors have a large effect on MEG responses during somatosensory^18^ and motor tasks^19^.

Using a source reconstruction technique for MEG signals, task-related cortical activities can be evaluated for each anatomical area. In particular, we have shown that changes in the signal power of neural oscillations, i.e. event-related synchronisation (ERS) and event-related desynchronisation (ERD)^20–22^, represent the cortical activity relevant to the task. Moreover, we have recently shown that genetic factors largely explain the alteration of frequency-specific cortical activity during word recognition^23^. Both for action recognition and action execution, β-band ERD was mainly enhanced in the sensorimotor and parietal areas^24–26^. Therefore, we hypothesised that the variations of β-band ERD during action observation, which are commonly observed during action execution, are due to genetic factors. In the present study, we quantified the effect of genetic and environmental factors on cortical activity related to action observation by comparing MEG responses of elderly monozygotic and dizygotic twins during the observation of hand movements.

## Results

### Action observation and task execution

**Figure 1:**
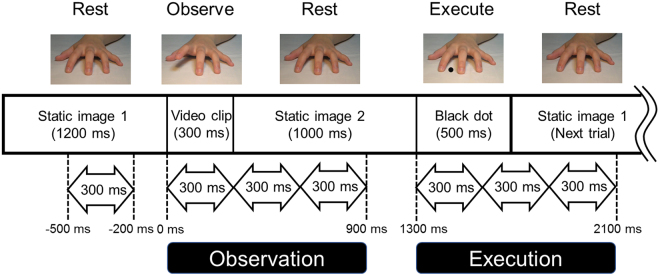
Schematic illustration of the task and analysis. After presenting a static picture of the hand for 1,200 ms, a video clip showing the movement of an index finger or middle finger being lifted was presented for 300 ms. Next, the same static picture was presented for 1,000 ms. Finally, a black dot was presented on the static picture for 500 ms. When the black dot appeared, the subjects were tasked with mirroring the finger movement from the video clip with the right hand. The start of the video clip was defined as 0 ms. The period from −500 ms to −200 ms was referred to as the resting period. MEG signals from 0 ms to 900 ms were analysed in three time windows of 300 ms each for action observation, and signals from 1,300 ms to 2,100 ms were analysed in three time windows of 300 ms each for action execution.

Twenty monozygotic twins and 11 dizygotic twins were enrolled in this study after obtaining written informed consent in accordance with the protocols approved by the ethics committee of the Osaka University Graduate School of Medicine. MEG signals were recorded for all subjects lying in the recumbent position on a bed in a magnetically shielded room. During the scan, the subjects were instructed to fix their gaze on the centre of the screen in front of them. After being presented with an image of a resting left hand for 1,200 ms, a video clip of the movement of the index or middle finger being lifted was presented for 300 ms. Thereafter, a black dot was presented on the image between the index and middle fingers as a signal for the subject to reproduce the movement. When this dot appeared, the subjects had to lift their own finger immediately to imitate the movement presented in the preceding video clip (Fig. 1).

### Neural oscillations during action observation and execution

**Figure 2:**
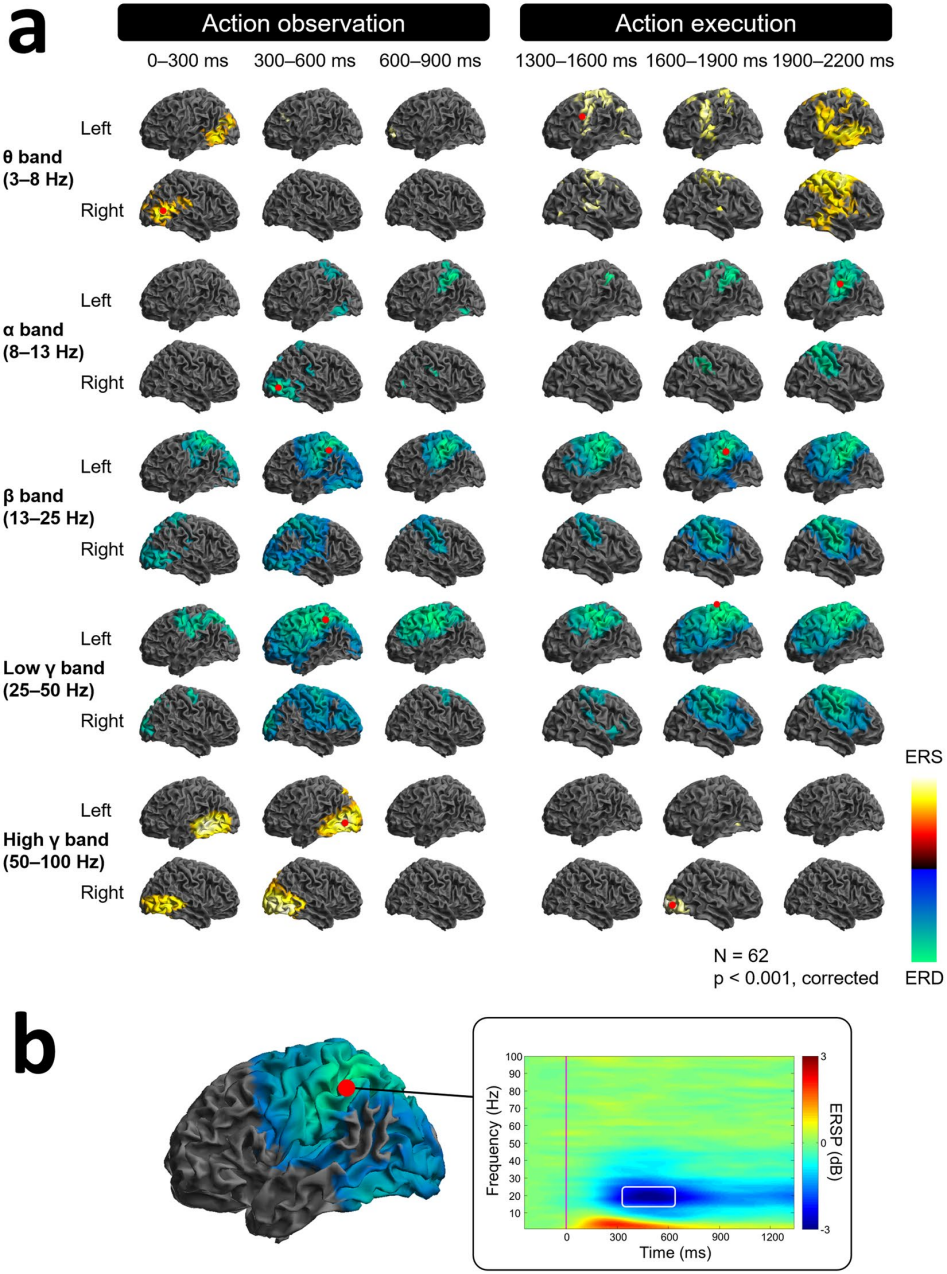
Neural oscillations during action observation and execution. (**a**) Spatiotemporal distributions of the oscillatory changes in 62 subjects (monozygotic and dizygotic twins) during action observation and execution. Only significant *t*-values of ERS/ERD were colour-coded on the normalised brain surface. The locations of the largest *t*-values are designated as red dots for each of the five frequency bands. (**b**) Grand averaged time–frequency spectrogram at the peak coordinates of the β-band ERD. ERSP = event-related spectral perturbation.

Significant ERS/ERD was observed during the observation and execution of hand movements. Figure 2 shows the spatiotemporal distribution of significant ERS/ERD based on the statistical analysis during action observation and execution. The peaks of the ERS/ERD are distributed widely, including the middle temporal gyrus (MTG), middle occipital gyrus (MOG), inferior temporal gyrus (IFG), inferior parietal lobule (IPL) and superior parietal lobule (SPL) (Fig. 2a, Tables 1,2). Moreover, the peak varied among frequency bands and with time after presentation of the stimulus. The red points in Fig. 2a show the spatiotemporal peaks of the significant ERS/ERD for each frequency band (Tables 1 and 2). Similarly, significant ERS/ERD was observed during action execution. Interestingly, the spatiotemporal peak of β-band ERD was also observed at the left IPL during the execution of an action. Notably, at the spatiotemporal peak in the left IPL, ERD was dominantly observed for the β-band (Fig. 2b). Therefore, commonly for the action observation and execution, the left IPL showed a significant ERD of the β-band.

**Table 1:** Peak coordinates of the largest *t*-value based on group statistical analysis during action observation.

Frequency band	Time (ms)	ERD/ERS	H	MNI coordinate^*^			*t*-value	Brain area
				X	Y	Z		
θ	0–300	ERS	R	50	−62	6	8.9	Middle temporal gyrus
α	300–600	ERD	R	44	−78	−2	9.9	Middle occipital gyrus
β	300–600	ERD	L	−32	−46	46	14.8	Inferior parietal lobule
Low γ	300–600	ERD	L	−42	−40	50	12.8	Inferior parietal lobule
High γ	300–600	ERS	L	−46	−76	2	9.8	Middle occipital gyrus

^*^Peak coordinates are on the grey matter.

MNI = Montreal Neurological Institute, H=hemisphere, L=left hemisphere, R=right hemisphere.

**Table 2:** Peak coordinates of the largest *t*-value based on group statistical analysis during action execution.

Frequency band	Time (ms)	ERD/ERS	H	MNI coordinate*			*t*-value	Brain area
				X	Y	Z		
θ	1,300–1,600	ERS	L	−70	−2	28	9.2	Precentral gyrus
α	1,900–2,200	ERD	L	−50	−28	34	9.5	Postcentral gyrus
β	1,600–1,900	ERD	L	−32	−44	42	14.7	Inferior parietal lobule
Low γ	1,600–1,900	ERD	L	−34	−26	74	13.2	Precentral gyrus
High γ	1,600–1,900	ERS	R	20	−100	−2	7.3	Cuneus

^*^Peak coordinates are on the grey matter.

MNI = Montreal Neurological Institute, H = hemisphere, L = left hemisphere, R = right hemisphere.

### Correlation of ERS/ERD between twins

**Figure 3:**
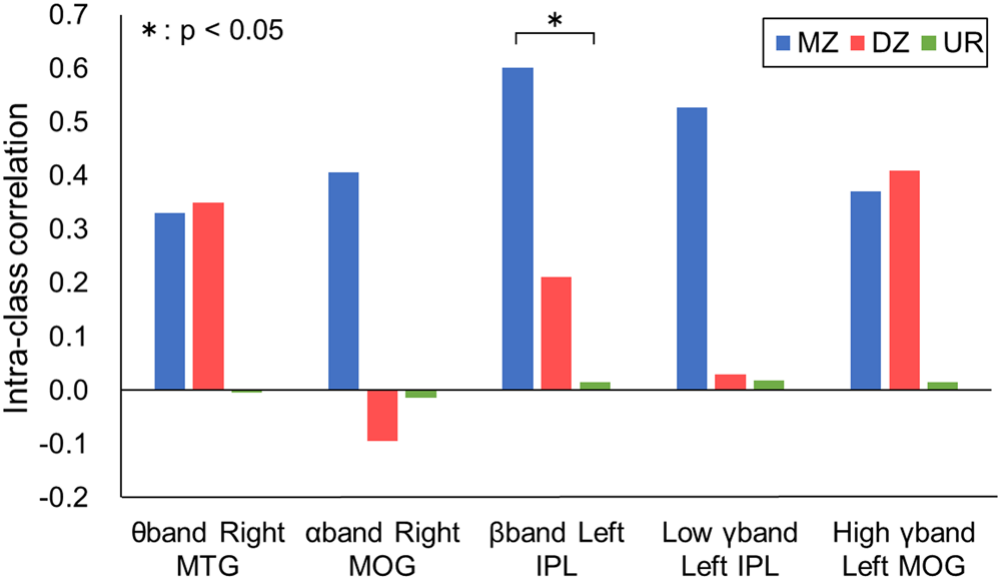
Intra-class correlations of ERSPs at the peak coordinates of five frequency bands were evaluated among monozygotic twins, dizygotic twins and unrelated pairs during action observation. An asterisk indicates a significant difference (Z-test, *p* < 0.05). MTG, middle temporal gyrus; MOG, middle occipital gyrus; IPL, inferior parietal lobule; M.Z., monozygotic twins; D.Z., dizygotic twins; U.R., unrelated pairs.

At the peak location of ERD/ERS among all subjects, the powers of each subject were compared among subject pairs in monozygotic and dizygotic twins and in unrelated pairs. The correlation coefficients of the powers between the pairs were evaluated for each of the three groups (intra-class correlations). Table 3 summarizes the intra-class correlations for each frequency band at each peak during three time windows of action observation. At 300–600 ms, the ERD of the β-band was significantly correlated among the monozygotic twin pairs (*p* = 0.036, Bonferroni-corrected, Fig. S1). The intra-class correlation of β-band ERD was significantly higher for monozygotic twins than for the unrelated pairs (Z-test, *p* = 0.027, Fig. 3). Also, for ERDs of the α-band, the intra-class correlations were significant at the right SPL for 300–600 ms (*p* = 0.00068, Bonferroni-corrected) and at the left IPL for 600–900 ms (*p* = 0.00048, Bonferroni-corrected) among monozygotic twins (Supplementary Table S1). These intra-class correlations among monozygotic twins were significantly higher than those of the unrelated pairs and dizygotic pairs (Z-test, *p* < 0.001). Notably, for other frequency bands of θ, low γ and high γ, the ERS/ERDs were not correlated among monozygotic twin pairs. The intra-class correlations were significantly high for the ERD of α and β bands among the monozygotic twins.

**Table 3:** Intra-class correlations and ACE modelling results at the peak coordinates of the largest *t*-value during action observation.

Frequency band	Time (ms)	ERS/ERD	H	MNI coordinate*			ICC			ACE modelling		
				X	Y	Z	MZ	DZ	UR	*a*	*c*	*e*
θ	0–300	ERS	R	50	−62	6	0.33	0.35	0.00	0.00	0.34	0.66
α	300–600	ERD	R	44	−78	−2	0.41	−0.10	−0.01	0.35	0.00	0.65
β	300–600	ERD	L	−32	−46	46	0.60	0.21	0.01	0.59	0.00	0.41
Low γ	300–600	ERD	L	−42	−40	50	0.53	0.03	0.02	0.49	0.00	0.51
High γ	300–600	ERS	L	−46	−76	2	0.37	0.41	0.02	0.00	0.38	0.62

^*^Peak coordinates are on the grey matter.

MNI = Montreal Neurological Institute, H = hemisphere, L = left hemisphere, R = right hemisphere, ICC = intra-class correlation. a = path coefficient for A (additive genetics), c = path coefficient for C (common environment), e = path coefficient for E (environment).

**Figure 4:**
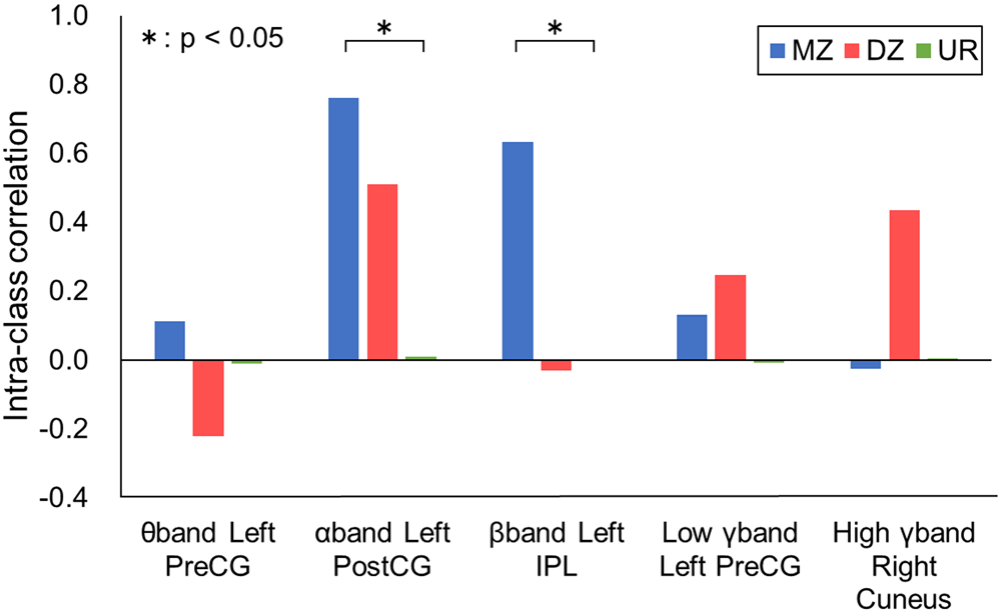
Intra-class correlations of ERSPs at the peak coordinates of five frequency bands were evaluated among monozygotic twins, dizygotic twins and unrelated pairs during action execution. An asterisk indicates a significant difference (Z-test, *p* < 0.05). PreC.G., precentral gyrus; PostC.G., postcentral gyrus; I.P.L., inferior parietal lobule; M.Z., monozygotic twins; D.Z., dizygotic twins; U.R., unrelated pairs.

Similarly, Fig. 4 shows the intraclass correlations of three groups during action execution. The intra-class correlation was significantly high for ERDs of the α-band at the left postcentral gyrus (postCG) (*p* = 0.00090, Bonferroni-corrected) and for ERD of the β-band at the left IPL (*p* = 0.022, Bonferroni-corrected) among monozygotic twins. The intra-class correlations among monozygotic twins were significantly higher than those of the unrelated pairs and dizygotic pairs (Z-test, *p* < 0.001).

In addition to intra-class correlation, we examined absolute differences of ERS/ERD power between the twin pairs during action observation and execution (Supplementary Tables S2 and S3). During action observation, the absolute difference in β-band ERD between monozygotic twin pairs was significantly smaller than that of unrelated pairs (*p* = 0.0091, Bonferroni-corrected). Other differences, in the θ-, α-, low γ- and high γ-bands, between monozygotic pairs tend to be smaller than those between dizygotic and unrelated pairs, but the difference is not significant. Also, during action execution, absolute differences of the α- and β-band ERDs between monozygotic twin pairs were significantly smaller than those of unrelated pairs (α-band ERDs: *p* = 0.000069, β-band ERDs: *p* = 0.0033).

### Heritability and genetic analysis of ERS/ERD

The relative contributions of additive genetic (A), common environmental (C) and environmental factors (E) were estimated through genetic structural equation modelling (Tables 3 and 4). During action observation, the heritability of β-band ERD at the left IPL was 67% (*a* = 0.59 and *e* = 0.41), which was higher than that of the other frequency bands: α-band ERD at the left MOG was 22% (*a* = 0.35 and *e* = 0.65) and low γ-band ERD at the left IPL was 49% (*a* = 0.49 and *e* = 0.51). During action execution, the heritability of α-band ERD at the left postcentral gyrus was 77% (*a* = 0.35, *c* = 0.19 and *e* = 0.65). The heritability of β-band ERD at the left IPL was 65% (*a* = 0.58 and *e* = 0.42).

**Table 4:** Intra-class correlations and ACE modelling results at the peak coordinates of the largest *t*-value during action execution.

Frequency band	Time (ms)	ERS/ERD	H	MNI coordinate*			ICC			ACE modelling		
				X	Y	Z	MZ	DZ	UR	*a*	*c*	*e*
θ	1300–1600	ERS	L	−70	−2	28	0.11	−0.22	−0.01	0.02	0.00	0.98
α	1900–2200	ERD	L	−50	−28	34	0.76	0.51	0.01	0.57	0.19	0.24
β	1600–1900	ERD	L	−32	−44	42	0.63	−0.03	0.00	0.58	0.00	0.42
Low γ	1600–1900	ERD	L	−34	−26	74	0.13	0.25	−0.01	0.00	0.22	0.78
High γ	1600–1900	ERS	R	20	−100	−2	−0.02	0.43	0.00	0.00	0.13	0.87

^*^Peak coordinates are on the grey matter.

MNI = Montreal Neurological Institute, H = hemisphere, L = left hemisphere, R = right hemisphere, ICC = intra-class correlation.

## Discussion

In this study, we identified significant ERSs and ERDs in each frequency band at various locations during action observation and execution. Among them, the β- and low γ-band ERDs at the IPL during action observation had higher similarity among monozygotic twins than among dizygotic twins and unrelated pairs. Similarly, the α-band ERD at the postcentral gyrus and the β-band ERD at the IPL during action execution had high similarity among monozygotic twins. These results demonstrate that genetic factors influence brain activity during action observation and execution depending on the frequency and spatial differences of the activities.

We observed significant ERSs/ERDs of the θ- and α-bands at the pre- and postcentral gyrus during action execution. These ERSs/ERDs are the cortical oscillatory changes involved in movement. In particular, the heritability of the powers was high for α-band ERD at the left postcentral gyrus (77%) and for β-band ERD at the left IPL (65%). In a previous twin study, we found more similarities in the latency and current source location of movement-evoked field 1 in monozygotic twins compared with unrelated pairs^19^. In the present study, we compared spatial and frequency patterns of MEG signals among monozygotic twins, dizygotic twins and unrelated pairs, to evaluate more precisely the genetic and environmental influences on movement-related cortical activities. By combining these results, we have evidence strongly supporting that movement-related cortical activities are affected by genetic influences.

It is noteworthy that the ERD of the left IPL is commonly observed both during action observation and execution in the β-band. It is known that the left IPL is part of the mirror neuron system^3,27^. This system contributes to the imitation of other people’s actions^5,28^ as well as to the perception of the intentions of others^4^. Thus, the task of imitating finger movements in this study may have activated the mirror neuron system in the left IPL. In addition, the heritability of β-band ERD powers at the left IPL was about 65% during both action observation and execution. This result suggests that the power of β-band ERD, which represents the activities of the mirror neuron system, is more influenced by genetic factors than by environmental factors.

Previous studies demonstrated that EEG powers are significantly correlated among monozygotic twins regardless of the cortical area^17,29^. However, the similarity in EEG signals may be due to the similarity of brain construction between twins (e.g. skull and cerebrospinal fluid) because EEG signals are influenced by differences in conductivity as determined by brain constructions. However, the MEG signal is theoretically not influenced by differences in conductivity. Moreover, the correlation was significant for the β-power band at the left IPL but not for other ERS/ERD. However, the other frequency band powers showed significant ERS/ERD in other cortical areas. Therefore, our results demonstrate that cortical activation during the cognitive task is influenced by genetic factors and depends on the cortical areas and frequency bands. The genetic influence is especially significant for the β power at the IPL during action observation.

Genes acting as genetic factors for the cortical activities have so far not been identified. A previous study has demonstrated that catechol-*O*-methyltransferase (COMT) Val/Met polymorphism was associated with variations in ERD/ERS responses using MEG during a working memory task^30^. In that study, Val homozygotes, as compared to Met homozygotes, increased ERD in the β-band in the left frontal-parietotemporal area. It is reasonable to hypothesise that such genetic factors influence the β-band ERD associated with the mirror neuron system.

Since we did not evaluate motor performance in this study, the relationship between brain oscillations and the performance of actual movement is unknown. In the future, to unveil the genetic factors of motor performance correlated with the cortical activities, detailed evaluation of the motor performance would be necessary.

Recently, we showed that the power of the low-γ ERD in the left frontal area during a language task was more homogeneous between monozygotic twins than between dizygotic twins^23^. However, the intra-class correlation of the β power at the left IPL during action observation in the present study (intra-class correlation of monozygotic twins = 0.60, intra-class correlation of dizygotic twins = 0.21) was larger than that of the previous study (intra-class correlation of monozygotic twins = 0.42, intra-class correlation of dizygotic twins = 0.24). These results indicate that cognitive function, such as action observation, is more innate than language function and is affected by genetics. The heritability of cortical activity varies depending on frequency, anatomical location and task. MEG studies involving twins can reveal the hereditary relationship between brain functions and the corresponding cortical activity. Our results demonstrate that genetic factors strongly affect the cortical activities for higher cognitive function.

It should be noted that the sample size of this study is smaller than in other twin studies. However, we have previously demonstrated that the cortical activities measured by MEG were highly correlated among twins during simple movement^19^ and language tasks^23^. In this study, the ERD during the observation of action was dominant for β bands (Fig. 2b). These results strongly support that the sample size of this study is sufficient to evaluate the particular MEG responses for the present task. Although the signal-to-noise ratio (SNR) might be improved with a larger sample, and this would allow showing the hereditability of other frequency bands, it might be difficult to evaluate the power changes of frequency bands higher than the β and low γ bands due to the low SNR in MEG signals. Our analysis of the hereditability of the β ERD was done with a sufficient SNR and sample size to detect genetic influences.

## Methods

### Subjects

Subjects participated voluntarily and registered with the Center for Twin Research at Osaka University^31^. We assessed 20 monozygotic twins (six males and 14 females; mean age, 58.1 ± 11.0 years) and 11 dizygotic twins (five males and six females; mean age, 61.4 ± 17.3 years). In all dizygotic twin pairs, both individuals of each twin pair were of the same sex. Zygosity was confirmed using the 15 loci of short tandem repeat markers derived from the blood^32^. Twin pairs that agreed completely for these short tandem repeat markers were designated as monozygotic, whereas all other pairs were designated as dizygotic.

All subjects demonstrated normal comprehension and intellectual capacity. All subjects were right-hand dominant per the Edinburgh Handedness Inventory^33^. Written informed consent was obtained from all subjects after an explanation of the purpose and possible consequences of the study. All experimental procedures were performed in accordance with the protocols approved by the ethics committee of the Osaka University Graduate School of Medicine (No. 10190). The study was conducted in accordance with the Ethical Guidelines for Medical and Health Research Involving Human Subjects issued by the Ministries of Education, Culture, Sports, Science and Technology (MEXT) and Health, Labour and Welfare (MHLW) in Japan.

### Measurements

A 160-channel whole-head neuromagnetometer device was used (MEG vision NEO; Ricoh Company, Ltd., Yokohama, Japan). Visual stimuli were displayed on a projection screen 325 mm from the subject’s eyes using a visual presentation system (Presentation; Neurobehavioral Systems, Berkeley, CA, USA) and a liquid-crystal projector (LVP-HC6800; Mitsubishi Electric Corporation, Tokyo, Japan).

The MEG data were recorded using an online band-pass filter between 0.01 and 200 Hz and sampled at 1,000 Hz. We also recorded electrooculograms and electrocardiograms (ECG) to remove the artefacts offline and surface electromyograms (EMG) of the right extensor indicis and extensor digitorum muscles to monitor finger movements. A 3D-T1 volume brain MRI was performed for all subjects using a 3.0-T magnetic resonance scanner.

A 3D digitiser (FastSCAN Corba, Polhemus, Colchester, VT, USA) and five head localisation coils attached to the subject’s head were used to align the MEG data with the individual brain MRI results. The data of the 3D digitiser were superimposed on the individual’s MRI data.

### Tasks

After the presentation of the resting left hand image for 1,200 ms, the subjects observed the video clip of the index finger or middle finger being lifted without moving their own fingers for 300 ms. The resting hand image was then presented again for 1,000 ms; thereafter, a black dot was presented on the image between index and middle finger. When this dot appeared, the subjects lifted their own finger immediately to emulate the movements presented in the preceding video clip (Fig. 1). Using EMG activity, we assessed whether the subject moved the correct finger. This sequence was repeated 70 times. The order in which to move either finger was randomized.

### Data analyses

MEG data were analysed using standard MEG software (MEG Laboratory; Ricoh Company, Ltd., Yokohama, Japan). We used an offline band-pass filter between 0.1 and 100 Hz and a band-elimination filter between 58 and 62 Hz (AC components). Eye blink artefacts on the data were separated and rejected by signal–space projection, a method for separating external disturbances, used in the Brainstorm software (version 3.2, http://neuroimage.usc.edu/brainstorm). Using signal–space projection, we detected obvious eye blinks from channels of the bilateral frontal base and rejected their artefacts from the data. We defined the onset time of the finger being lifted as 0 ms. The time window of a trial was defined as −500 to 2,100 ms. Among the 70 trials for each subject, unsuitable trials that included excessive muscle activity were not used in the analyses.

### Beamformer

To calculate ERS/ERD, the data of each subject were analysed using a narrow-band adaptive beamformer, which is a spatial filtering method to improve the spatial resolution of neuromagnetic sources^34^. We used the beamformer software (coohea; Ricoh Company, Ltd., Yokohama, Japan). Estimates of differences in the source power between baseline and the period of interest for the selected time windows and frequency bands were computed as pseudo-*t*-values^20^. The distribution of the pseudo-*t*-values was then superimposed onto the individual anatomical MRI results coregistered to the MEG data. Beamformer created a volume that covered the whole brain of each subject with a voxel size of 5 × 5 × 5 mm^3^. The baseline was from −500 to −200 ms. The periods of interest during action observation were three 300-ms windows totalling 0 to 900 ms (0–300, 300–600 and 600–900 ms). The periods of interest during action execution were three 300-ms windows totalling 1,300 to 2,100 ms (1,300–1,600, 1,600–1,900 and 1,900–2,100 ms). The MEG data were divided into θ- (3–8 Hz), α- (8–13 Hz), β- (13–25 Hz), low γ- (25–50 Hz) and high γ- (50–100 Hz) bands by fast Fourier transformation (FFT).

### Group statistical analysis

The cortical areas commonly activated among subjects were identified by group statistical maps. We used the statistical parametric mapping software package SPM8 (http://www.fil.ion.ucl.ac.uk/spm/) to examine the statistical significance of ERS/ERD across subjects. The coregistered MRI data were normalised to the Montreal Neurological Institute (MNI) template with a voxel size of 2 × 2 × 2 mm^3^. A one-sample *t*-test at each voxel level was performed using a *t*-value incorporating variance smoothing with a 10-mm Gaussian kernel width. A *p*-value of < 0.001 (family-wise error rate) in each voxel was considered a statistically significant difference. Significant voxels were superimposed onto the MNI template brain using SPM8. We analysed only the surface voxels of the brain.

### Virtual sensors and time–frequency analysis

After identifying the spatiotemporal peaks of significant ERS/ERD during action observation using group statistical analysis, the cortical currents at the peak location in each frequency band were estimated by a virtual sensor toolbox (BRAM; Ricoh Company, Ltd., Yokohama, Japan). Next, a time–frequency analysis was applied to the estimated cortical currents.

The virtual sensor projects the signals exclusively from the targeted voxel using the adaptive beam-forming technique^35^. The beamformer is constructed to project signals exclusively from targeted voxels while eliminating residual noise to suppress signals from other parts of the brain. Thus, the virtual sensors provide data regarding neural activity at the target voxels with a considerably higher SNR than that of raw MEG data. The virtual sensor data were estimated by the individual MEG data divided into θ (3–8 Hz), α (8–13 Hz), β (13–25 Hz), low γ (25–50 Hz) and high γ (50–100 Hz) bands by FFT.

First, we determined the peak coordinates of the largest *t*-value for each frequency band using group statistical analysis. Next, the selected coordinate was used as the target location for the virtual sensor. The virtual sensor’s coordinates were extracted from individual MRI results based on the MNI coordinates and the warping parameters were calculated by SPM8 using an MRI-T1 template and individual MRI-T1 images. A tomographic reconstruction of the data was created by generating a single-sphere head model based on the head shape obtained from the structural MRI results for each subject.

A time–frequency analysis was applied to these signals to determine the oscillatory changes at the location of the virtual sensor in each subject, which was performed using FFT in the EEGLAB software (version 12.0.2.0, https://sccn.ucsd.edu/eeglab/) with MATLAB [version 8.2.0.701 (R2013b); The MathWorks Inc.]). Event-related spectral perturbations (ERSPs) were calculated while the window of 0.511 s overlapped 400 points (every 0.4 s) with the 1000 Hz sampling. Values at each time–frequency point were averaged over the trials. The baseline period extended from −500 to −200 ms. The frequency range of 1–100 Hz and the data windows were zero-padded with a ratio of 4. During the observation of action, the average power of ERSPs was calculated for each of the five frequency bands for each estimated cortical current from the 0- to 900-ms poststimulus time window with three 300-ms intervals. In the same way, during action execution, the average power of ERSPs was calculated from the 1,300- to 2,100-ms poststimulus time window with three 300-ms intervals.

### Calculation of correlation coefficients and genetic analysis

To compare the similarities in the power of ERS/ERD within twin pairs from both groups (monozygotic and dizygotic twins), we calculated the intra-class correlation between ERS/ERD power for the members of each twin pair and unrelated pairs. To apply the ACE model, the averages and variances of ERS/ERD power in twin1 (*x* axis) and twin2 (*y* axis) should not be significantly different^36^. We, therefore, randomly selected twin1 and twin2 so that there was no significant difference in the average and variance value of ERS/ERD power in twin1 and twin2 pairs. The group of 27 unrelated pairs was constructed by randomly selecting 54 subjects from all subjects of the same sex with age differences within 5 years. The unrelated pairs were generated 100 times to evaluate the intra-class correlation. Next, the intra-class correlations were averaged to estimate the intra-class correlation of the sex- and age-matched unrelated pairs. In addition, we calculated absolute differences of ERS/ERD power between the twin pairs. We compared these absolute differences among monozygotic or dizygotic twins to the differences of all unrelated pairs from each group (363 pairs in monozygotic twins and 124 pairs in dizygotic twins of same sex).

The genetic influences on individual differences were estimated by an ACE model to the powers of ERS/ERD in which the observed variance of the phenotype of interest is decomposed into genetic and environmental components. These components include variance caused by additive genetics effects (A), common environmental effects (C) and individual environmental effects including measurement error (E). The relative contribution of these latent factors is expressed by the path coefficients *a*, *c* and *e*, which are estimated by maximum likelihood. To estimate the values of *a*, *c* and *e*, the ERS/ERD data of the monozygotic and dizygotic twin pairs were analysed using the OpenMX program (http://openmx.psyc.virginia.edu/) and R statistical software.

## Electronic supplementary material

Supplementary information

## References

[1] Gallese V, Fadiga L, Fogassi L, Rizzolatti G (1996). Action recognition in the premotor cortex. Brain..

[2] Rizzolatti G, Fadiga L, Gallese V, Fogassi L (1996). Premotor cortex and the recognition of motor actions. Brain Res. Cogn. Brain Res..

[3] Rizzolatti G, Fogassi L, Gallese V (2001). Neurophysiological mechanisms underlying the understanding and imitation of action. Nature Rev. Neurosci..

[4] Iacoboni M (2005). Grasping the intentions of others with one’s own mirror neuron system. PLoS Biol..

[5] Kessler K (2006). Investigating the human mirror neuron system by means of cortical synchronization during the imitation of biological movements. Neuroimage..

[6] Wicker B (2003). Both of us disgusted in my insula: the common neural basis of seeing and feeling disgust. Neuron..

[7] Spilka MJ, Steele CJ, Penhune VB (2010). Gesture imitation in musicians and non-musicians. Exp. Brain Res..

[8] Meriau K (2006). A neural network reflecting individual differences in cognitive processing of emotions during perceptual decision making. Neuroimage..

[9] Wirth M, Villeneuve S, La Joie R, Marks SM, Jagust WJ (2014). Gene-environment interactions: lifetime cognitive activity, APOE genotype, and beta-amyloid burden. J. Neurosci..

[10] Calvo-Merino B, Glaser DE, Grezes J, Passingham RE, Haggard P (2005). Action observation and acquired motor skills: an FMRI study with expert dancers. Cereb. Cortex..

[11] Smit DJ, Posthuma D, Boomsma DI, Geus EJ (2005). Heritability of background EEG across the power spectrum. Psychophysiol..

[12] Begleiter H, Porjesz B (2006). Genetics of human brain oscillations. Int. J. Psychophysiol..

[13] Ahveninen J (2006). Inherited auditory-cortical dysfunction in twin pairs discordant for schizophrenia. Biol. Psychiatry..

[14] van Pelt S, Boomsma DI, Fries P (2012). Magnetoencephalography in twins reveals a strong genetic determination of the peak frequency of visually induced gamma-band synchronization. J. Neurosci..

[15] Pinel P, Dehaene S (2013). Genetic and environmental contributions to brain activation during calculation. Neuroimage..

[16] Moodie CA, Wisner KM, MacDonald AW (2014). Characteristics of canonical intrinsic connectivity networks across tasks and monozygotic twin pairs. Hum. Brain Mapp..

[17] van Beijsterveldt CE, Molenaar PC, de Geus EJ, Boomsma DI (1996). Heritability of human brain functioning as assessed by electroencephalography. Am. J. Hum. Genet..

[18] Van’t Ent D, Van Soelen IL, Stam KJ, De Geus EJ, Boomsma DI (2010). Genetic influence demonstrated for MEG-recorded somatosensory evoked responses. Psychophysiol..

[19] Araki T (2014). Genetic and environmental influences on motor function: a magnetoencephalographic study of twins. Front. Hum. Neurosci..

[20] Hirata M (2004). Determination of language dominance with synthetic aperture magnetometry: comparison with the Wada test. Neuroimage..

[21] Piai V, Roelofs A, Rommers J, Maris E (2015). Beta oscillations reflect memory and motor aspects of spoken word production. Hum. Brain Mapp..

[22] Sacchet MD (2015). Attention drives synchronization of alpha and beta rhythms between right inferior frontal and primary sensory neocortex. J. Neurosci..

[23] Araki T (2016). Language-related cerebral oscillatory changes are influenced equally by genetic and environmental factors. Neuroimage.

[24] Muthukumaraswamy SD, Johnson BW, Gaetz WC, Cheyne DO (2006). Neural processing of observed oro-facial movements reflects multiple action encoding strategies in the human brain. Brain Res..

[25] Muthukumaraswamy SD, Singh KD (2008). Modulation of the human mirror neuron system during cognitive activity. Psychophysiol..

[26] Sugata H (2017). Frequency-dependent oscillatory neural profiles during imitation. Sci Rep..

[27] Rizzolatti G, Craighero L (2004). The mirror-neuron system. Annu. Rev. Neurosci..

[28] Buccino G (2004). Neural circuits underlying imitation learning of hand actions: an event-related fMRI study. Neuron.

[29] van Beijsterveldt CE, van Baal GC (2002). Twin and family studies of the human electroencephalogram: a review and a meta-analysis. Biol. Psychol..

[30] Altamura M (2016). The impact of Val108/158Met polymorphism of catechol-O-methyltransferase on brain oscillations during working memory. Neurosci. Lett..

[31] Hayakawa K, Iwatani Y (2013). & Osaka Twin Research, G. An overview of multidisciplinary research resources at the Osaka University Center for Twin Research. Twin Res. Hum. Genet..

[32] Yang MJ, Tzeng CH, Tseng JY, Huang CY (2006). Determination of twin zygosity using a commercially available STR analysis of 15 unlinked loci and the gender-determining marker amelogenin–a preliminary report. Hum. Reprod..

[33] Oldfield RC (1971). The assessment and analysis of handedness: the Edinburgh inventory. Neuropsychologia..

[34] Dalal SS (2008). Five-dimensional neuroimaging: localization of the time-frequency dynamics of cortical activity. Neuroimage..

[35] Sekihara K, Nagarajan SS, Poeppel D, Marantz A, Miyashita Y (2002). Application of an MEG eigenspace beamformer to reconstructing spatio-temporal activities of neural sources. Hum. Brain Mapp..

[36] Rijsdijk FV, Sham PC (2002). Analytic approaches to twin data using structural equation models. Brief Bioinform..

