# Supplementary material for: Frequency-specific genetic influence on inferior parietal lobule activation commonly observed during action observation and execution
Source: Sci Rep. 2017 Dec 15;7:17660. doi: 10.1038/s41598-017-17662-x (PMC5732255; doi:10.1038/s41598-017-17662-x)
Supplement: Supplementary file 1 — Supplementary information [file 41598_2017_17662_MOESM1_ESM.docx]

Supplementary Information for

Frequency-specific genetic influence on inferior parietal lobule activation commonly observed during action observation and execution

Toshihiko Araki^1, 2^, Mai Onishi^1^, Takufumi Yanagisawa^1, 3, 4^, Masayuki Hirata^3, 4^, Yoshiyuki Watanabe^5^, Soshiro Ogata^6,7^, Kazuo Hayakawa^8^, Chika Honda^9^, Mikio Watanabe^9, 10^, Yoshinori Iwatani^9, 10^ and Shiro Yorifuji^1^

^1^Division of Functional Diagnostic Science, Graduate School of Medicine, Osaka University, Osaka 565-0871, Japan

^2^Department of Medical Technology, Osaka University Hospital, Suita, Osaka, 565-0871, Japan

^3^Division of Clinical Neuroengineering, Global Center for Medical Engineering and Informatics, Osaka University, Osaka 565-0871, Japan

^4^Department of Neurosurgery, Graduate School of Medicine, Osaka University, Osaka 565-0871, Japan

^5^Department of Diagnostic and Interventional Radiology, Graduate School of Medicine, Osaka University, Osaka 565-0871, Japan

^6^Department of Health Promotion Science, Graduate School of Medicine, Osaka University, Osaka 565-0871, Japan

^7^Research Fellow of Japan Society for the Promotion of Science, Tokyo, Japan

^8^Mie Prefectural College of Nursing, Tsu, Mie 514-0116, Japan

^9^Center for Twin Research, Graduate School of Medicine, Osaka University, Osaka 565-0871, Japan

^10^Division of Biomedical Informatics, Graduate School of Medicine, Osaka University, Osaka 565-0871, Japan

**Contents:**

Supplementary Table 1—3

Supplementary Figure 1

**Table S1.** Intra-class correlation and ACE modelling showing significant ERS/ERDs at all peak coordinates during action observation. Asterisk indicates significant correlation (*p* < 0.05, Bonferroni-corrected). The peak coordinates of the largest *t*-value are shown in bold type.

| Frequency band | Time (ms) | ERS/  ERD | H | MNI coordinate | | | *t*-value | Brain area | ICC | | | ACE-model | | |
| --- | --- | --- | --- | --- | --- | --- | --- | --- | --- | --- | --- | --- | --- | --- |
|  |  |  |  | X | Y | Z |  |  | MZ | DZ | UR | a | c | e |
| **θ** | **0–300** | **ERS** | **R** | **50** | **−62** | **6** | **8.85** | **Middle temporal gyrus** | **0.33** | **0.35** | **0.00** | **0.00** | **0.34** | **0.66** |
|  |  |  | L | 0 | **−**76 | 4 | 8.82 | Lingual gyrus | **−**0.16 | 0.41 | 0.00 | 0.00 | 0.04 | 0.96 |
|  | 600–900 |  | L | **−**32 | 58 | 0 | 7.53 | Superior frontal gyrus | 0.11 | **−**0.35 | 0.02 | 0.02 | 0.00 | 0.98 |
| **α** | **300–600** | **ERD** | **R** | **44** | **−78** | **−2** | **9.89** | **Middle occipital gyrus** | **0.41** | **−0.10** | **−0.01** | **0.35** | **0.00** | **0.65** |
|  |  |  | R | 56 | **−**24 | 20 | 7.42 | Postcentral gyrus | 0.25 | **−**0.22 | **−**0.02 | 0.18 | 0.00 | 0.82 |
|  |  |  | L | **−**46 | **−**58 | **−**12 | 7.39 | Middle occipital gyrus | 0.04 | **−**0.50 | 0.00 | 0.00 | 0.00 | 1.00 |
|  |  |  | R | 16 | **−**42 | 74 | 7.19 | Postcentral gyrus | 0.59 | 0.34 | 0.02 | 0.48 | 0.10 | 0.41 |
|  |  |  | L | **−**38 | **−**50 | 56 | 7.03 | Inferior parietal lobule | 0.60 | 0.14 | 0.00 | 0.58 | 0.00 | 0.42 |
|  |  |  | L | **−**30 | **−**38 | 68 | 6.94 | Postcentral gyrus | 0.49 | 0.39 | 0.01 | 0.16 | 0.32 | 0.52 |
|  |  |  | R | 24 | **−**74 | 44 | 6.46 | Superior parietal lobule | 0.78* | 0.03 | -0.01 | 0.77 | 0.00 | 0.23 |
|  | 600–900 |  | L | **−**48 | **−**38 | 42 | 8.47 | Inferior parietal lobule | 0.79* | 0.32 | 0.02 | 0.79 | 0.00 | 0.21 |
|  |  |  | L | **−**28 | **−**34 | 66 | 6.94 | Postcentral gyrus | 0.49 | 0.50 | 0.03 | 0.00 | 0.49 | 0.51 |
|  |  |  | R | 36 | **−**40 | 38 | 7.35 | Inferior parietal lobule | 0.47 | 0.26 | 0.04 | 0.40 | 0.06 | 0.54 |
|  |  |  | R | 60 | **−**18 | 22 | 7.19 | Postcentral gyrus | 0.28 | 0.16 | 0.00 | 0.22 | 0.06 | 0.72 |
|  |  |  | L | **−**54 | **−**70 | **−**14 | 6.93 | Middle occipital gyrus | 0.01 | **−**0.17 | 0.00 | 0.00 | 0.00 | 1.00 |
|  |  |  | R | 36 | **−**68 | 2 | 6.83 | Middle occipital gyrus | 0.20 | **−**0.18 | **−**0.03 | 0.13 | 0.00 | 0.87 |
|  |  |  | R | 36 | **−**48 | **−**4 | 6.3 | Parahippocampal gyrus | 0.36 | 0.03 | **−**0.03 | 0.33 | 0.00 | 0.67 |
| β | 0–300 | ERD | L | **−**32 | **−**48 | 50 | 10.49 | Inferior parietal lobule | 0.24 | **−**0.11 | 0.03 | 0.18 | 0.00 | 0.82 |
|  |  |  | L | **−**28 | **−**40 | 62 | 9.96 | Postcentral gyrus | 0.25 | **−**0.31 | 0.04 | 0.15 | 0.00 | 0.85 |
|  |  |  | L | **−**12 | **−**32 | 58 | 9.72 | Medial frontal gyrus | 0.26 | 0.06 | 0.03 | 0.25 | 0.00 | 0.75 |
|  | **300–600** |  | **L** | **−32** | **−46** | **46** | **14.8** | **Inferior parietal lobule** | **0.60*** | **0.21** | **0.01** | **0.59** | **0.00** | **0.41** |
|  | 600–900 |  | L | **−**46 | **−**32 | 46 | 12.06 | Inferior parietal lobule | 0.58 | 0.42 | 0.01 | 0.29 | 0.29 | 0.43 |
|  |  |  | R | 44 | **−**24 | 40 | 9.15 | Postcentral gyrus | 0.31 | **−**0.13 | 0.02 | 0.25 | 0.00 | 0.75 |
|  |  |  | L | **−**58 | **−**6 | 58 | 9 | Precentral gyrus | 0.50 | 0.46 | 0.01 | 0.05 | 0.45 | 0.51 |
| Low γ | 0–300 | ERD | L | **−**4 | **−**68 | 48 | 9.19 | Precuneus | 0.13 | 0.11 | 0.03 | 0.03 | 0.10 | 0.87 |
|  |  |  | L | **−**28 | **−**60 | 52 | 8.49 | Superior parietal lobule | 0.18 | **−**0.25 | 0.00 | 0.11 | 0.00 | 0.89 |
|  |  |  | L | **−**54 | **−**24 | 34 | 8.28 | Postcentral gyrus | 0.47 | 0.11 | 0.00 | 0.45 | 0.00 | 0.55 |
|  |  |  | R | 34 | **−**16 | 60 | 7.74 | Precentral gyrus | **−**0.30 | 0.19 | **−**0.03 | 0.00 | 0.00 | 1.00 |
|  | **300–600** |  | **L** | **−42** | **−40** | **50** | **12.75** | **Inferior parietal lobule** | **0.53** | **0.03** | **0.02** | **0.49** | **0.00** | **0.51** |
|  |  |  | L | **−**58 | **−**26 | 40 | 12.35 | Postcentral gyrus | 0.26 | 0.13 | 0.03 | 0.25 | 0.01 | 0.75 |
|  |  |  | L | **−**54 | **−**10 | 56 | 12.21 | Precentral gyrus | 0.46 | 0.42 | 0.00 | 0.06 | 0.40 | 0.54 |
|  | 600–900 |  | L | **−**50 | **−**10 | 62 | 10.15 | Precentral gyrus | 0.35 | 0.01 | 0.00 | 0.31 | 0.00 | 0.69 |
|  |  |  | L | **−**52 | **−**22 | 38 | 9.47 | Postcentral gyrus | 0.48 | 0.21 | 0.01 | 0.47 | 0.00 | 0.53 |
|  |  |  | L | **−**34 | **−**16 | 70 | 9.31 | Precentral gyrus | 0.37 | **−**0.30 | 0.00 | 0.27 | 0.00 | 0.73 |
| High γ | 0–300 | ERS | R | 50 | **−**46 | 0 | 7.89 | Middle temporal gyrus | **−**0.24 | **−**0.07 | 0.00 | 0.00 | 0.00 | 1.00 |
|  | **300–600** |  | **L** | **−46** | **−76** | **2** | **9.75** | **Middle occipital gyrus** | **0.37** | **0.41** | **0.02** | **0.00** | **0.38** | **0.62** |
|  |  |  | R | 34 | **−**92 | 0 | 9.56 | Middle occipital gyrus | 0.07 | 0.24 | 0.03 | 0.00 | 0.13 | 0.87 |

ERS = event-related synchronisation, ERD = event-related desynchronization, MNI = Montreal Neurological Institute, ICC = intra-class correlation, MZ = monozygotic twins, DZ = dizygotic twins, UR = unrelated pairs.

**Table S2.** Absolute differences of ERS/ERD powers between twin pairs at the peak coordinates of the largest *t*-value during action observation. Asterisk indicates the absolute difference between twin pairs is significant smaller than that of unrelated pairs (*p* < 0.05, Bonferroni-corrected).

| Frequency band | Time (ms) | ERS/ERD | H | MNI coordinate | | |  | ERS/ERD difference  [Mean (SD)] | |
| --- | --- | --- | --- | --- | --- | --- | --- | --- | --- |
|  |  |  |  | X | Y | Z |  | MZ | DZ |
| θ | 0–300 | ERS | R | 50 | −62 | 6 |  | 1.22 (0.71) | 1.00 (0.77) |
| α | 300–600 | ERD | R | 44 | −78 | −2 |  | 1.09 (0.87) | 1.91 (1.24) |
| β | 300–600 | ERD | L | −32 | −46 | 46 |  | 0.93 (0.62)* | 1.26 (1.18) |
| Low γ | 300–600 | ERD | L | −42 | −40 | 50 |  | 0.43 (0.36) | 0.67 (0.51) |
| High γ | 300–600 | ERS | L | −46 | −76 | 2 |  | 0.14 (0.17) | 0.27 (0.20) |

MNI = Montreal Neurological Institute, H = hemisphere, L = left hemisphere, R = right hemisphere, MZ = monozygotic twins (20 pairs), DZ = dizygotic twins (11 pairs). Absolute difference indicates average and variance value in each pair.

**Table S3.** Absolute differences of ERS/ERD powers between twin pairs at the peak coordinates of the largest *t*-value during action execution. Asterisk indicates the absolute difference between twin pairs is significantly smaller than that of unrelated pairs (*p* < 0.05, Bonferroni-corrected).

| Frequency band | Time (ms) | ERS/ERD | H | MNI coordinate | | | ERS/ERD difference (Mean (SD)) | |
| --- | --- | --- | --- | --- | --- | --- | --- | --- |
|  |  |  |  | X | Y | Z | MZ | DZ |
| θ | 1300-1600 | ERS | L | −70 | −2 | 28 | 0.94 (0.61) | 1.34 (0.90) |
| α | 1900-2200 | ERD | L | −50 | −28 | 34 | 0.75 (0.73)* | 1.59 (1.04) |
| β | 1600-1900 | ERD | L | −32 | −44 | 42 | 0.95 (0.66)* | 1.89 (1.23) |
| Low γ | 1600-1900 | ERD | L | −34 | −26 | 74 | 0.58 (0.46) | 0.78 (0.59) |
| High γ | 1600-1900 | ERS | R | 20 | −100 | −2 | 0.29 (0.25) | 0.35 (0.42) |

MNI = Montreal Neurological Institute, H = hemisphere, L = left hemisphere, R = right hemisphere, MZ = monozygotic twins (20 pairs), DZ = dizygotic twins (11 pairs). Absolute difference indicates mean and standard deviation value in each pair.


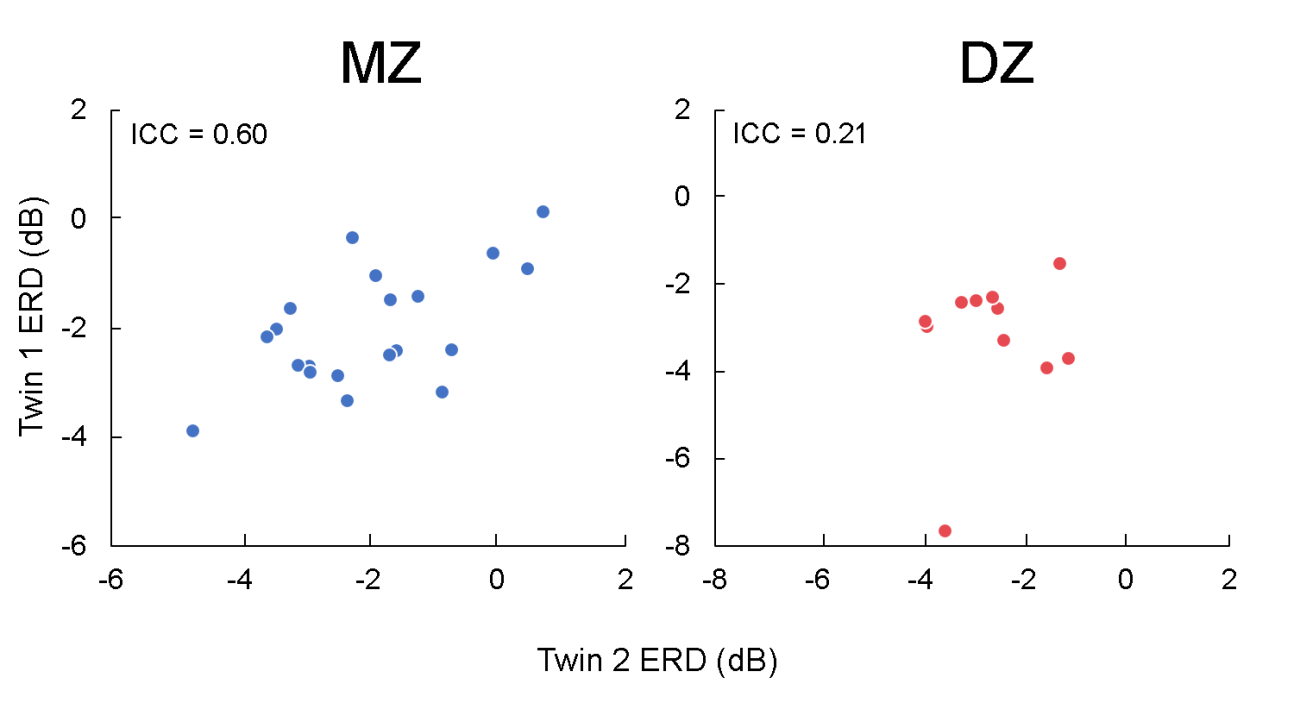


**Figure S1.** Correlation between the powers of the β-band ERD at the IPL in the 300–600-ms post-stimulus window. Each data point represents the power of one twin versus that of their co-twin. MZ, monozygotic twins; DZ, dizygotic twins; ICC, intra-class correlation.
